# Federated Learning-Based CNN Models for Orthodontic Skeletal Classification and Diagnosis

**DOI:** 10.3390/diagnostics15070920

**Published:** 2025-04-02

**Authors:** Demet Süer Tümen, Mehmet Nergiz

**Affiliations:** 1 Department of Orthodontics, Faculty of Dentistry, Dicle University, 21280 Diyarbakır, Türkiye; 2 Department of Computer Engineering, Dicle University, 21280 Diyarbakır, Türkiye; mnergiz@dicle.edu.tr

**Keywords:** federated learning, convolutional neural network, orthodontic skeletal classification, cephalometric images, DenseNet121, spatial attention

## Abstract

**Background/Objectives:** Accurate skeletal classification is essential for orthodontic diagnosis. This study evaluates the effectiveness of federated convolutional neural network (CNN) models for skeletal classification using cephalometric images from the ISBI and Dicle datasets. This research aims to evaluate the effectiveness of federated learning (FL) for orthodontic skeletal classification by comparing its performance against centralized learning (CL) and local learning (LL). The objective is to determine whether FL can achieve competitive performance while preserving data privacy and enabling collaborative model training across multiple institutions. **Methods:** The DenseNet121 model and its augmented versions, incorporating channel attention, spatial attention, squeeze and excitation, and spatial pyramid pooling blocks, are proposed and adapted for the study. Models are evaluated on the ISBI and Dicle datasets using accuracy, sensitivity, and specificity metrics, with performance gains benchmarked across CL, LL, and FL frameworks. **Results:** Accuracy improvements exceed 26% compared to the baseline model on FL framework. The DenseNet121_SA model, augmented with spatial pyramid pooling blocks, achieves a 20.86% performance gain over LL settings on the ISBI dataset. Similarly, the DenseNet121_SA model, augmented with spatial attention, and DenseNet121_SA_SE model, augmented with spatial attention and squeeze and excitation, obtain 16.58% and 15.22% by not sacrificing performance loss with respect to CL. The inclusion of the Dicle dataset provides additional validation for the models. **Conclusions:** Federated CNN models exhibit significant promise for orthodontic skeletal classification. These models demonstrate the potential of FL to enhance collaborative model training while preserving data privacy. This approach represents a step forward in leveraging precise orthodontic diagnostics technology by enabling a data-secure collaborative artificial intelligence among various orthodontic clinics.

## 1. Introduction

**Figure 1:**
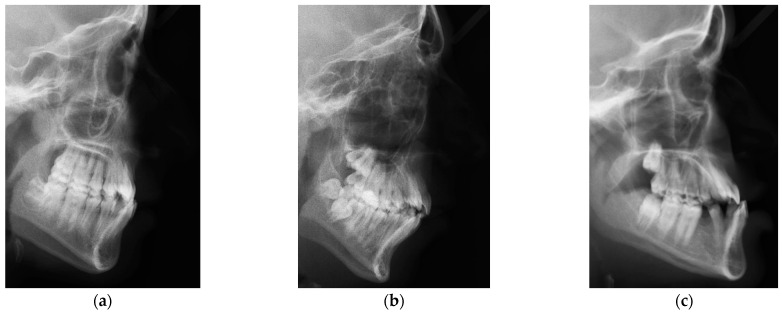
Cropped images representing Class I, II, and III categories from Dicle dataset: (**a**) Class I; (**b**) Class II; (**c**) Class III.

Clinical applications of computerized automatic dental radiography analysis systems offer efficiencies by reducing time and manual expenses as well as inter- and intra-observer inconsistencies [1]. In orthodontics, the classification of skeletal patterns is a standard practice for diagnosing conditions and formulating treatment strategies [2]. Accurate diagnosis holds clinical significance as it impacts treatment planning and outcomes. The orthodontic skeletal classification task concentrates on examining the positional connections between the lower and upper jaws relative to a stationary reference point. For instance, Steiner’s classification system relies on the alignment of three specific anatomical landmarks, which are the A, N, and B points, to determine the ANB angle [2]. Considering the ANB angle, three categories are utilized to establish classifications for skeletal patterns—I, II and III—as shown in Figure 1 [2,3]. Class I exhibits a typical anteroposterior alignment between the jaws and often features a normal-looking facial profile. Class II is characterized by a posterior positioning of the mandible in relation to the maxilla and usually displays a facial profile that curves outward. Class III demonstrates an anterior relationship between the upper jaw and mandible and typically exhibits a facial profile that curves inward [2,3].

Deep learning (DL) and more specifically convolutional neural network (CNN)-based studies on orthodontics face two primary challenges [4]: Firstly, automating the detection of anatomical landmarks by AI models is a prevalent diagnostic challenge [4]. Secondly, employing CNN algorithms directly for cephalometric image-based classification or analysis presents another common challenge [4]. In contrast to the automated tracing AI model, the second approach, which is preferred in this study also, can bypass the need for landmark detection and interpretation of cephalometric measurements [4].

Developing effective and resilient AI applications for dentistry demands extensive and top-notch datasets, often scattered across various sources such as disparate clinical institutes [5]. In contrast to other medical domains, the availability of top-notch datasets for orthodontic research is restricted and the collaborative endeavors face restrictions due to privacy concerns [6]. At this critical point, federated learning (FL) offers a scalable and privacy-conscious approach to collaborative AI model training by facilitating knowledge exchange derived from the data without the need for data sharing [5]. FL allows numerous contributors to collectively train AI models, expanding access to insights from a broader range of diverse sensitive data sources without the need for direct data sharing [5]. Nevertheless, the use of FL in dental research remains relatively unexplored [5,7,8]. The fact that there are currently very few published works about applying FL on dentistry images inspired us to initiate this research.

### Literature Review

Ibragimov et al. developed a skull landmark detection framework using random forests and Haar-like features, achieving up to 76.64% accuracy on the ISBI dataset [1]. Lindner and Cootes introduced an automated landmark detection system (RFRV-CLM) with random forest regression and shape optimization, achieving up to 75.83% accuracy [9]. Nino-Sandoval et al. used SVM classifiers to distinguish skeletal classes based on craniomaxillary variables, achieving 74.51% accuracy [2]. While two-phase methods offer better performance, they rely on precise annotation of 19 landmarks per image in new datasets. In contrast, the approach in this study aims to require only class labels during training. Moreover, this approach is expected to reduce labeling efforts by using just three landmark annotations per image, which could significantly lessen the data preparation workload for large datasets in the future.

Kim et al. evaluated various CNN architectures for predicting orthognathic surgery needs, with ResNet-18 achieving the highest AUC of 0.979 [10]. Kim et al. also proposed a simple CNN for sagittal skeletal classification on cephalometric images, reaching 96% accuracy and high sensitivity and specificity [4]. These two related studies also applied the class label of the whole image like the proposed study. However, these works were applied only on their own local datasets and there is still room to apply the whole-image approach using different models on different datasets. Moreover, none of the aforementioned methods applied the FL approach, which is applied to each proposed model in this study.

The aim of this research is to investigate the application of federated CNN models utilizing cephalometric images of two distinct imaging datasets for performing orthodontic skeletal classification by using only the region of interest (ROI) of the whole image directly, without the need of landmark annotations during the testing phase. This research provides the following contributions:

1. DenseNet121 and five other improved novel models are transformed into their federated architectures through the utilization of the Flower FL framework and the skeletal classification is performed without the need of landmark annotations;
2. This study, based on our understanding, marks the initial instance of orthodontic skeletal classification in the literature conducted in a federated manner, presenting a unique aspect of this work;
3. The Dicle dataset, comprising cephalometric imaging data, is made publicly available;
4. The impact of FL is thoroughly examined using two distinct dental datasets—the IEEE International Symposium on Biomedical Imaging 2015 Cephalometric X-ray Image Analysis Challenge (ISBI 2015) and Dicle datasets—as a detailed analysis of FL’s contribution is crucial for advancing further clinical applications.

The organization of this article is outlined as follows: The Section 1 states the big picture of the aim of this research, which applies the FL approach to orthodontic skeletal classification problems. The Section 2 provides information regarding the two datasets utilized in this research and elaborates on the proposed CNN models and their FL implementations. The Section 3 presents the performance outcomes of various settings on the proposed models. The Section 4 evaluates the level of achievements through FL against the other settings and compares them with existing studies. The Section 5 encapsulates the findings of the study.

## 2. Materials and Methods

### 2.1. ISBI Dataset

A dataset comprised of lateral cephalogram radiographs was constructed for the ISBI 2015—abbreviated as ISBI in this article—dental image analysis grand challenge [1,11]. Permission to perform the study was granted by the research ethics committee of the Tri-Service General Hospital in Taipei, Taiwan, under IRB Number 1-102-05-017 [1]. The dataset comprises lateral cephalometric radiographs in two dimensions gathered from 400 distinct patients, totaling 400 images [11]. The patients’ statistical characteristics are as follows: their ages span from 7 to 76 years, with a mean age of 27 years [11]. A total of 235 of the ISBI dataset are females, whereas the remaining 165 are males. The images are captured utilizing the Soredex CRANEX^®^ Excel Ceph machine from Tuusula, Finland. Each image has a pixel resolution of 1935 × 2400 pixels in .bmp format, with a pixel size of 0.1 mm. The ground truth for assessing the locations of 19 landmarks in all of the images is established through manual marking and inspection by two experts.

**Figure 2:**
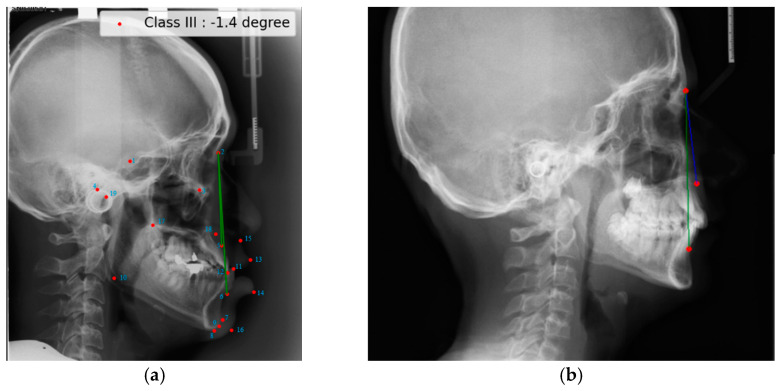
The orthodontic skeletal class labeling tools in the ISBI and Dicle datasets: (**a**) auto-labeling by ANB angle in ISBI; and (**b**) Class II by manual labeling ANB angle (5.6) in Dicle.

In this study, the class labels of the images of the ISBI dataset are decided by automatically measuring the ANB angle via a simple piece of code in which A, N and B points are handled as the 5th, 2nd, and 6th landmarks, respectively, as shown in Figure 2a. During the labeling procedure, the Steiner analysis is set at 3.2–5.7 for Class 1, exceeding 5.7 for Class 2, and falling below 3.2 for Class 3, mirroring the approach taken by Ibragimov et al. [1]. Additionally, the results of that automatic labeling are verified by an experienced orthodontist. The obtained data distribution of the ISBI is given in Table 1.

### 2.2. Dicle Dataset

This article introduces a new public imaging dataset, referred to as Dicle, which consists of lateral cephalogram radiographs. These images are sourced from anonymized retrospective records obtained from the Department of Orthodontics at Dicle University. Approval to conduct the research was obtained from the research ethics committee of the Dicle University Faculty of Dentistry, Türkiye, with IRB Number 2023-13. All methodologies adhered to relevant guidelines and regulations. The dataset contains lateral cephalometric radiographs in two dimensions collected from 856 individual patients, resulting in 856 images. The demographic profile of the patients is as follows: Their ages range from 9 to 46 years, with an average age of 17.8 years. Out of the 856 patients, 499 are female and 357 are male. Only patients who underwent orthodontic diagnosis at the Department of Orthodontics at Dicle University, Türkiye, between March 2019 and March 2023 are included in this study. The images were captured using the Planmeca Promax machine from Helsinki, Finland. The images vary in pixel resolution, with some having a resolution of 1942 × 2175 pixels in .jpg format and others having a resolution of 1676 × 2175 pixels. The ground truth for evaluating the positions of three (ANB) landmarks in all images of Dicle dataset is established through manual marking and examination by an experienced orthodontist, as shown in Figure 2b. In the process of labeling, Class 1 is designated with a Steiner analysis ranging from 0 to 4, surpassing 4 for Class 2, and dropping below 0 for Class 3, reflecting the method employed by Nino-Sandoval et al. and Kim et al. [2,4,10]. The images of both of the datasets are cropped manually by only covering the frontal region of the face. Three representative cropped images from Dicle dataset are selected for Class I, II, and III categories as shown in Figure 1.

In this study, the high number of Class I of Dicle dataset cases may be due to the increase in preventive and interceptive orthodontic approaches, rising interest in orthodontic treatment, and heightened awareness among parents in recent years, such as between March 2019 and March 2023, may have reduced the proportion of Class II and Class III patients. The majority of the subjects in Dicle dataset are of Turkish and Asian origin.

### 2.3. DenseNet121

The Dense Convolutional Network (DenseNet) establishes connections between each layer in a progressive manner, wherein each layer receives input from all preceding layers and transmits its own output to all subsequent layers [12]. This structure presents several benefits, including addressing the vanishing-gradient problem, enhancing the propagation of features, encouraging the reuse of features, and notably decreasing the number of parameters. To maintain the feed-forward flow, each layer receives inputs from previous layers and transmits its own output to subsequent layers. This design decreases parameter redundancy compared to traditional convolutional networks, thus necessitating fewer parameters. Additionally, dense connections act as a regularization mechanism, decreasing overfitting particularly in scenarios with limited training data. In this study, DenseNet121 based models, a specific variant within the DenseNet family, are employed.

**Figure 3:**
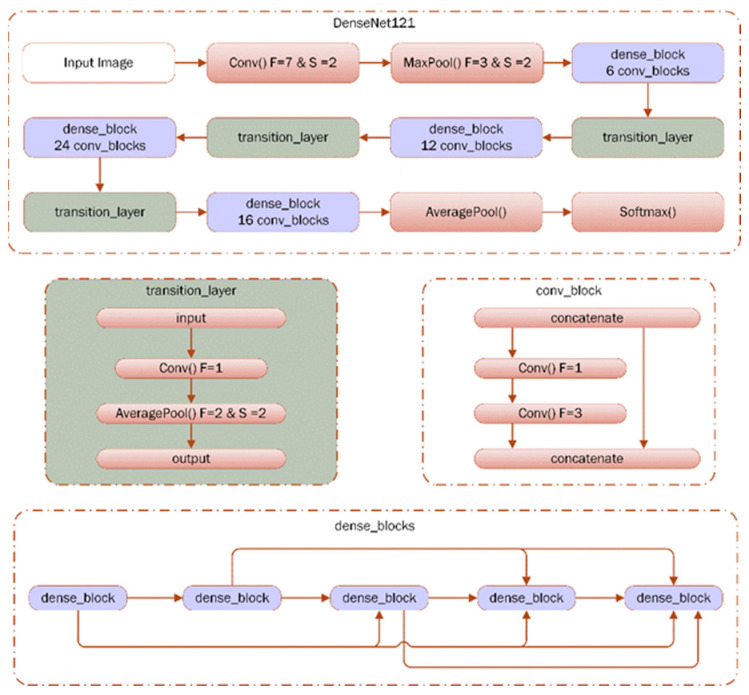
The structure of the DenseNet121 model [12,13].

The DenseNet121 implementation comprises three types of blocks. First of them is “convolution block”, serving as the fundamental unit within the “dense block” structure. The second type is the “dense block”, characterized by densely connected convolution blocks that are concatenated together, forming the core component of DenseNet121 [13]. Finally, the third block type is the “transition layer”, which bridges two neighboring dense blocks. Since the feature map sizes are constant within dense blocks, the transition layer’s role is to decrease the dimensions of the feature maps. All blocks employ a bottleneck design technique. The structure of DenseNet121 is depicted in Figure 3 where F and S symbolizes the convolutional filter and stride sizes, respectively.

### 2.4. Channel Attention

Woo et al. introduced the convolutional block attention module (CBAM), a straightforward yet potent attention mechanism designed for feed-forward CNNs [14]. Operating on an intermediate feature map, the CBAM module sequentially generates channel attention (CA) and spatial attention (SA) maps along distinct dimensions. These attention maps are then applied to the input feature map, enhancing its adaptability and refinement as well as prioritizing significant features while dampening irrelevant ones. Thanks to its lightweight and versatile nature, CA and SA seamlessly and independently integrate into various CNN architectures with minimal additional computational burden, offering end-to-end trainability alongside base CNNs. CA and SA enable complementary attention, pinpointing ‘what’ and ‘where’ aspects, respectively. In this study, CA and SA are appended to DenseNet121 model as independent modules.

Initially, Woo et al. consolidate spatial details within a feature map, denoted as F, by employing both average pooling and max-pooling techniques [14]. These descriptors are subsequently directed to a unified network aimed at generating the CA map. This shared network comprises a multi-layer perceptron (MLP) with one hidden layer. After applying the shared network to each descriptor, the resulting feature vectors are merged through element-wise addition, followed by the application of a sigmoid function. The computation of channel attention is formulated in Equation (1).

(1) $$A_{c}\left(F\right)=\sigma (MLP\left(AvgPool\left(F\right)\right)+MLP\left(MaxPool\left(F\right)\right))$$

### 2.5. Spaital Attention

To compute the spatial attention, the feature map F undergoes average-pooling and max-pooling operations along its channel axis [14]. These obtained pooling results are concatenated to generate a streamlined feature descriptor. This concatenated feature descriptor undergoes processing by a convolution layer, employing a 7 × 7 filter size, to obtain a two-dimensional spatial attention map indicating regions to highlight or dampen. Subsequently, the resultant spatial attention map is subjected to a sigmoid function. To conclude, the computation of spatial attention formulated and visualized as in Equation (2).

(2) $$A_{s}\left(F\right)=\sigma (f_{7\times 7}([AvgPool(F);MaxPool(F)]))$$

### 2.6. Squeeze and Excitation (SE)

The SE block is an architectural component which basically exploits the relationship between channels [15]. The SE block adaptively modifies feature responses on a per-channel basis by explicitly capturing correlations between channels. To implement feature recalibration using the SE block, the squeeze and excitation steps which are respectively formulated as Sq() and Ex() functions in Equations (3) and (4) are performed. Initially, features F undergo a compression operation, consolidating feature maps across spatial dimensions H × W via a global pooling process to produce a channel descriptor z. This descriptor captures the comprehensive distribution of feature responses across channels, enabling the integration of information from the network’s broader receptive field into its lower layers. Afterward, an excitation process occurs, wherein individual activations are learned for each channel through a self-gating mechanism involving fully connected (FC) layers as well as RELU and sigma activation functions dependent on channel relationships, regulating channel excitation. Afterward, an excitation process occurs where individual activations are learned for each channel through a self-gating mechanism. This mechanism involves fully connected (FC) layers, as well as ReLU and sigma activation functions, which regulate channel excitation based on channel relationships. The feature maps F are subsequently recalibrated to generate the SE block’s output by undergoing multiplication with the obtained $S_{c}$ scaling factor, which can then be directly input into subsequent layers of the main CNN. The mathematical formulations for the squeeze and excitation steps are provided in Equations (3) and (4), respectively.

(3) $$z_{c}=Sq\left(F_{c}\right)=\frac{1}{H\times W}\sum_{i=1}^{H}\sum_{j=1}^{W}F_{c}(i,j)$$

(4) $$S_{c}=Ex(z_{c})=\sigma (FC(RELU(FC(z_{c})))$$

### 2.7. Spatial Pyramid Pooling (SPP)

The SPP layer aggregates features and produces outputs of fixed length, which are then fed into the FC layers [16]. Essentially, the SPP conducts information “aggregation” at a deeper level of the network hierarchy, eliminating the need for initial cropping or warping and enabling the network to analyze feature maps in a multiscale manner. The SPP retains spatial details through pooling within local spatial bins. These bins are sized proportionally to the image dimensions, ensuring a fixed number of bins regardless of image size. To accommodate deep networks for images of varying sizes, the last pooling layer before the FC layer is substituted with an SPP layer. Within each spatial bin, the responses of each filter undergo pooling, with adaptive average pooling being utilized in this investigation. The outputs of the SPP consist of vectors with dimensions determined by the product of the number of spatial bins and the pooling sizes utilized. Specifically, the number of spatial bins corresponds to the number of filters in the final convolution layer. In this study, the pooling sizes are set as [4,8,16]. Following the implementation of the SPP, the obtained fixed-dimensional vectors are inputted into the FC layer.

**Figure 4:**
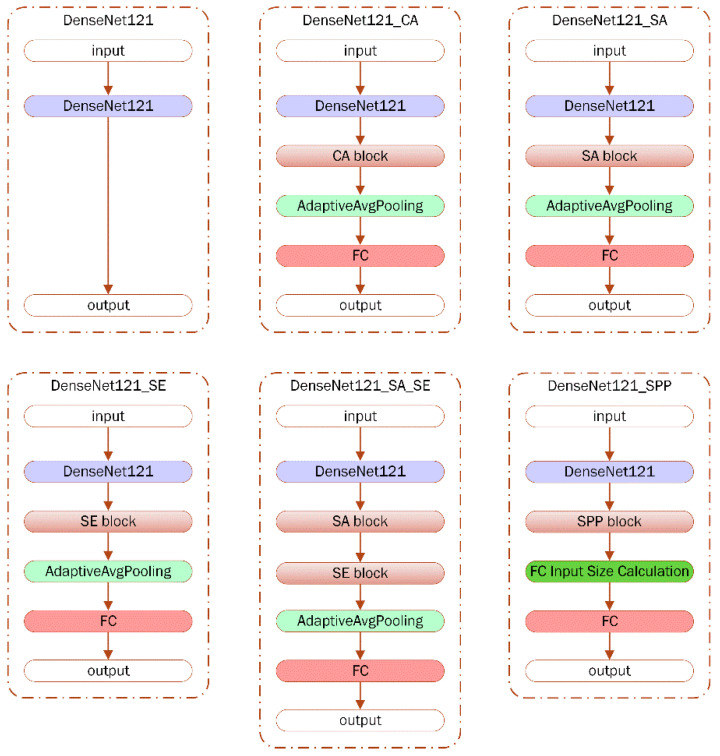
The main architectures of DenseNet121-based models of this study.

The main architectures of this study are set by appending the CA, SA, SE and SPP block to the end of the DenseNet121 model and 5 new augmented models are proposed as DenseNet121_CA, DenseNet121_SA, DenseNet121_SE, DenseNet121_SA_SE, DenseNet121_SPP as shown in Figure 4. In all of the augmented models, the FC part of the original DenseNet121 model is detached and the final feature map is connected to the following block as an input. Additionally, the result of the appended blocks is applied to adaptive average pooling before the final FC layer of the DenseNet121_CA, DenseNet121_SA, DenseNet121_SE and DenseNet121_SA_SE models. To mediate the tensor sizes of the results of the SPP block and the final FC layer, the “FC input size calculation” step is appended only in the DenseNet121_SPP architecture.

### 2.8. Setting Federated Learning for Dicle and ISBI Datasets

Google introduced the FL concept as an approach to developing ML models capable of learning from individual local datasets dispersed across numerous devices, all without the need to access data from other peers [17,18]. Various hospitals or research institutions have the opportunity to work together to enhance the strength of a ML model by leveraging FL technology, all while maintaining data privacy. The FL architecture, which is based on a client and server setup, simply necessitates the local training of the model by client sites, followed by iterative sharing with the server [19]. FL additionally offers a chance to distribute the workload of storage and computation among the clients [20].

**Figure 5:**
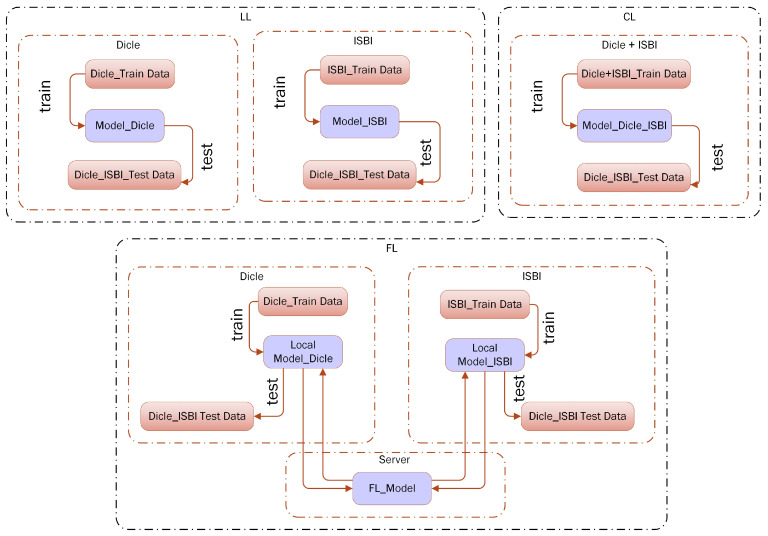
The LL, CL, and FL training and testing datasets and general training and testing flow.

In this study, the various DL methods are primarily implemented across three distinct settings, namely LL, CL, and FL. The suggested settings stem from diverse scenarios of data and model sharing among collaborating clients, characterized in this research as medical institutions possessing private cephalometric images. The local learning (LL) setting denotes a scenario where each collaborating client independently trains its unique model using its local data, while a shared test dataset is utilized by both clients. The centralized learning (CL) setting presents a scenario where both collaborators merge their data onto a single server, aiming to achieve the highest attainable accuracy. The FL setting entails a scenario where each collaborating client retains its individual local data, and the ultimate global model is derived solely through the iterative exchange of model parameter updates with a central server. The LL, CL, and FL training and testing datasets as well as the general training testing approach are depicted in Figure 5.

**Figure 6:**
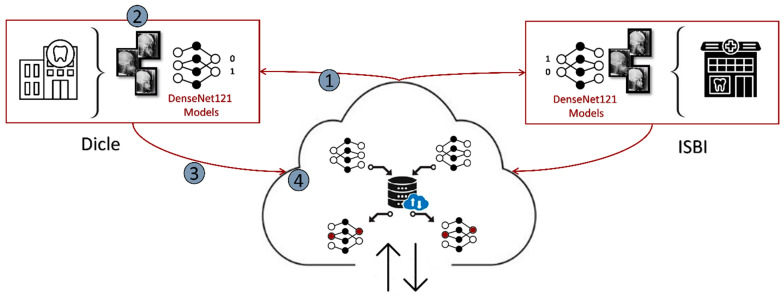
The FL settings of the Dicle and ISBI datasets.

As depicted in Algorithm 1 and Figure 6, the FL process primarily comprises four successive key stages [18]: Initially, the initial DL model is transmitted to the Dicle and ISBI clients, each of which possesses its own unique local dataset. At the onset of the initial round, the model initially dispatched is loaded onto the clients and trained with their respective local datasets. The weight updates from these trained models are then transmitted back to the server, where they are aggregated using the FedAvg algorithm. Subsequently, the aggregated model is once again forwarded to the clients for the subsequent round, continuing until the predetermined number of rounds is achieved. In this research, six distinct models—DenseNet121, DenseNet121_CA, DenseNet121_SA, DenseNet121_SE, DenseNet121_SA_SE, and DenseNet121_SPP—are adapted to the FL framework and evaluated.

In this study, the number of epochs for local training in both CL and LL settings is standardized at 100. For the FL setting, the local training epochs are set to 5, while the global training epochs are set to 50. All six models were constructed using PyTorch 2.0.1, and the code implementations for federated learning are developed and set up on Flower, a FL platform built on PyTorch [21]. The Flower platform additionally provides implementations of the SecAgg and SecAgg+ secure model aggregation algorithms, catering to the privacy-preserving needs of FL [21,22,23]. The experiments are conducted on a workstation equipped with dual Nvidia RTX A4000 16 GB GPUs, an Intel i7 3.6 GHz CPU, and 64 GB of RAM. The batch size is configured to 64, and the images are resized to 224 during both training and testing phases. The CNN weights are pre-trained using the ImageNet1K dataset. The optimizer is selected as Adam with a learning rate of 0.001 and the used loss function is CrossEntropLoss. The batch size is set as 64 and the input size is set as 224 for all the models except for inceptionV3, which requires an input size of 299. The applied data augmentation methods are RandomResizedCrop(input_size), RandomHorizontalFlip(), and Normalize() during the training phase. The data augmentation methods applied during the testing phase are Resized(256), CenterCrop(input_size), and Normalize().
**Algorithm 1**: The Algorithm of the FL setting on the Dicle and ISBI datasets**define:**  1.a: Client_i_, 1 ≤ i ≤ 2   1.b: LocalDataset_i_         // Local Dataset of Client_i_
  1.c: GlobalModel_itr_, itr = 0       // The Global DL Model initialized in Server, itr: global iteration number **start:** **do while**              // 50 global iterations for this study   1.d: Send(GlobalModel_itr_)        // Send the most recent version of the Global DL model at the itrth iteration  2: Train(GlobalModel_itr_, LocalDataset_i_) → (GlobalModel_itr_)_i_
  // Each ith client trains the loaded model with its local data for 5 local epochs for this study  3: **for each** Client_i_, **do**   SendServer((GlobalModel_itr_)_i_) → (GlobalModel_itr_)_server_
   //The obtained parameter updates of all the locally trained models are sent back to the server   4.a: FaultTolerantFedAvg (GlobalModel_itr_) → GlobalModel_itr_
  //The parameter updates are aggregated on the server and   //then a combined Global DL model is obtained for ith iteration   4.b: increment(itr) **end**

## 3. Results

### 3.1. Selecting the Baseline Model

In this study, two distinct cephalometric imaging datasets, namely Dicle and ISBI, are curated specifically for orthodontic skeletal classification tasks and are trained and tested on CL, LL, and FL settings. In order to determine the baseline model, a paired t-test is conducted by performing a five-fold cross-validation after verifying normality using the Shapiro–Wilk test. The obtained *p*-value results indicate that DenseNet121 is significantly better than ShuffleNet (*p* < 0.05), while differences with the other models—VGG, InceptionV3, and AlexNet—are not statistically significant. However, it is observed that DenseNet121 has the highest t-value, and thus the highest mean, for ACC on both datasets and the highest AUC for the ISBI dataset when DenseNet121 is compared with all the other models as listed in Table 2. An increasing body of evidence, alongside the statistical analysis results, also highlights that DenseNet121 has been successfully applied in a previous study for sagittal skeletal classification of children using cephalometric images, making it a reasonable choice for the baseline model [24]. During the step to decide the baseline model, the sophisticated models like ConvMixer are also benchmarked, but it is observed that it tends to classify all the images as Class II. Thus, the DenseNet121 model is selected as a baseline and all further modular augmentations are added to the basic model. Finally, the following augmented models are obtained: DenseNet121_CA, DenseNet121_SA, DenseNet121_SE, DenseNet121_SA_SE, and DenseNet121_SPP.

In the tables presented below, solely the outcomes characterized by the highest ACC values and their associated AUC-ROC values are listed. These AUC-ROC values are derived as the mean of the area under the curve calculations for individual classes within the datasets, employing the “one-vs-rest” methodology.

### 3.2. LL, CL and FL Results

Both the standard and augmented versions of DenseNet121 are trained and evaluated on Dicle and ISBI imaging datasets for LL, CL, and FL structures, with the outcomes detailed in Table 3, Table 4 and Table 5. The results for CL settings of basic and the augmented DenseNet121 models are given in Table 3. As stated before, the training and testing sets of both datasets are merged in the CL setting. The highest ACC value is obtained by DenseNet121_SE model in the CL setting. Furthermore, it is noteworthy to emphasize that the augmented models demonstrate superior performance compared to the standard DenseNet121 model, with an improvement of at least 22%.

All the proposed DenseNet based models are trained for the LL setting also. In this setting, the testing images of both of the datasets are merged but the training images of the datasets are kept independently local. The training and testing procedures are also performed locally for each of these datasets. The dominance of the DenseNet121_SE model is still observed in the LL setting of ISBI dataset, as shown in Table 4. However, the DenseNet121_SA_SE model gets the highest ACC in the LL setting of Dicle dataset.

The outcomes pertaining to FL setting for both the standard and augmented versions of DenseNet121 are outlined in Table 5. As previously indicated, within the FL setting, the training and testing datasets are stored locally and processed independently. The DenseNet121_SA_SE model achieves the highest ACC value. The precision, recall, and F1 score values in Table 5 are computed using the “weighted” averaging method in the sklearn library. Moreover, it is important to highlight the superior performance of augmented models over the standard DenseNet121, with an improvement of at least 26%.

**Figure 7:**
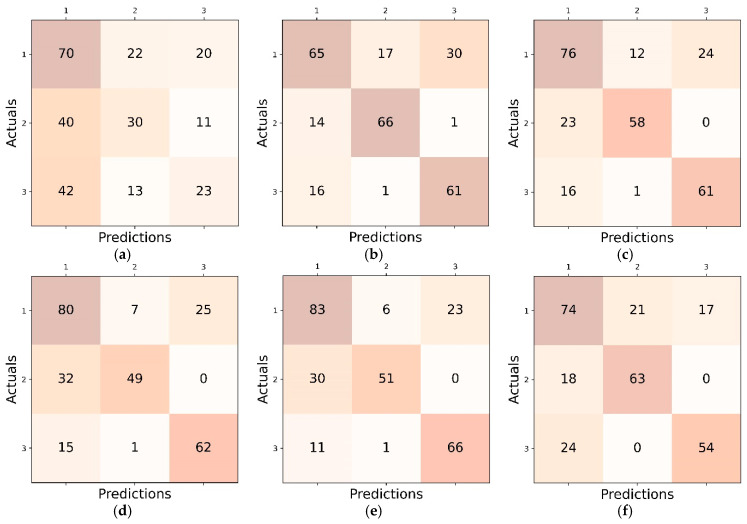
The confusion matrices illustrating classification performance in the FL setting: (**a**) DenseNet121; (**b**) DenseNet121_CA; (**c**) DenseNet121_SE; (**d**) DenseNet121_SA; (**e**) DenseNet121_SA_SE; and (**f**) DenseNet121_SPP.

The confusion matrices depicted in Figure 7 provide a detailed breakdown of classification results within the FL setting. Every matrix illustrates the occurrences of true positives, true negatives, false positives, and false negatives among three classes, providing an understanding of the model’s effectiveness and areas that could be enhanced.

**Figure 8:**
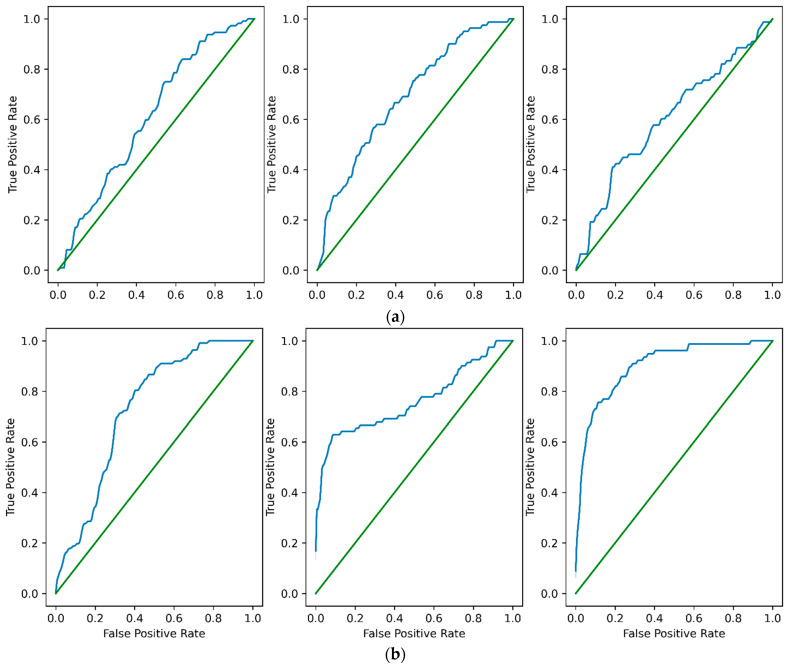
The AUC–ROC curves showing the performances of the models in the FL setting: (**a**) Class I, II, and III of DenseNet; and (**b**) Class I, II and III of DenseNet121_SA_SE.

Figure 8 showcases the AUC-ROC curves of DenseNet121 and DenseNet121_SA_SE tailored to the FL setting. Analyzing the AUC-ROC curves aids in assessing the model’s discriminatory ability across various thresholds and allows for comparisons between different FL configurations or algorithms.

The generalization capacity of a model can probably be measured best from the LL setting of the model, as it is trained on local training dataset but tested on the merged testing dataset. In the light of this evaluation approach, the DenseNet121_SA_SE and DenseNet121_SA_SE models have the highest generalization capacities, respectively, on the Dicle and ISBI datasets, as can be seen in Table 4. However, DenseNet121_SPP obtains the lowest performance, relatively, among the augmented DenseNet121 models.

### 3.3. FL Contribution with Respect to LL and CL

In this study, significant improvements are observed in accuracy through FL compared to LL settings, especially on the ISBI dataset, as its size is less than half that of the Dicle dataset. The contributions of FL to the LL setting and the amount of performance sacrifice from the CL setting based on the difference in the mean ACC values of the models across both datasets are outlined in Table 6. The FL setting shows better performance than the CL setting for the DenseNet121_SA and DenseNet121_SA _SE models, as can be seen in Table 6.

In Table 6, the highest accuracy contribution of FL over LL on the Dicle dataset and the lowest sacrifice of FL over CL on the ISBI dataset are obtained by the DenseNet121_SA_SE model, whereas the highest accuracy contribution of the ISBI dataset is obtained by the DenseNet121_SPP model. In Table 6, it can also be observed that the contribution amount of FL with respect to LL is higher and the performance sacrifice amount of FL with respect to CL is lower for the augmented models compared to the basic DenseNet121 model. This observation can be evaluated as the positive effect of FL is more apparent on the augmented models.

The superiority of DenseNet121_SA and DenseNet121_SA_SE models is noticeable, as FL obtains higher ACC values than CL. The highest sacrifice in accuracy performance of FL over CL is observed in the basic DenseNet121 model.

### 3.4. Model Convergence Analysis in FL Setting

**Figure 9:**
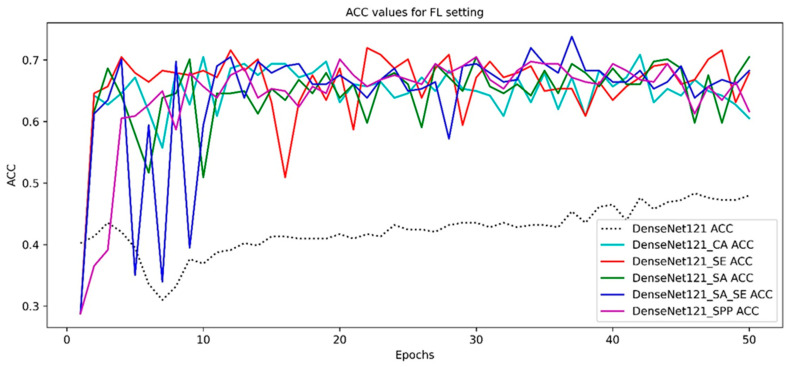
The convergence of the ACC curves of the models in the FL setting.

The convergence of the accuracy curves of the models are given through the epochs axis in Figure 9. The DenseNet121 ACC curve has the lowest but stably increasing pattern. The DenseNet121_CA is less volatile than the DenseNet121_SE. The DenseNet121_SA_SE is more volatile than the other two models but has the highest ACC peak value.

## 4. Discussion

### 4.1. Inter Class Performance Analysis in LL, CL and FL

**Figure 10:**
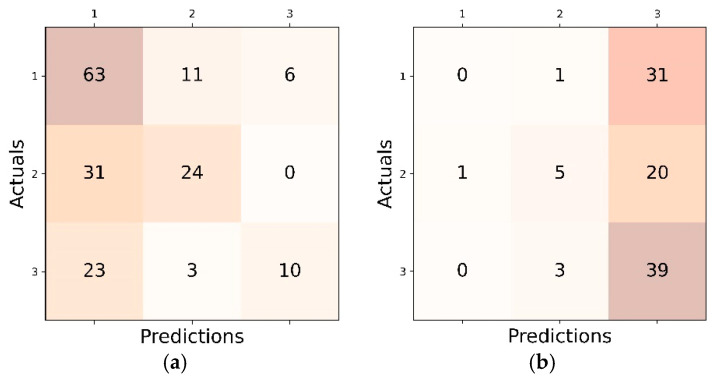

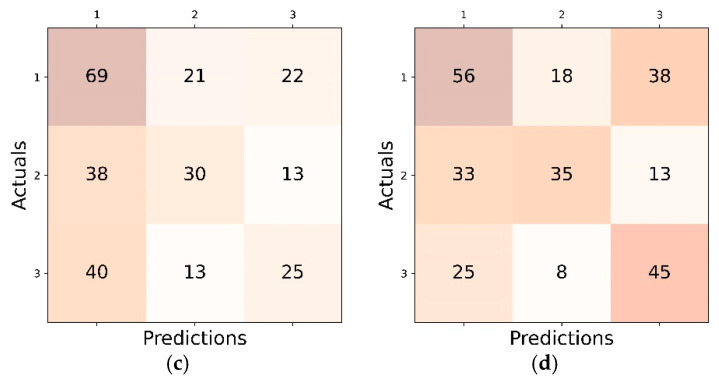
The confusion matrices models of DenseNet121 in the LL, FL, and CL settings. (**a**) the confusion matrix of DenseNet121 on Dicle dataset in the LL setting, (**b**) the confusion matrix of DenseNet121 on ISBI dataset in the LL setting, (**c**) the confusion matrix of DenseNet121 in the FL setting, (**d**) the confusion matrix of DenseNet121 in the CL setting.

The class distributions of both datasets are shown in the last row of Table 1. The dominant classes in the Dicle and ISBI datasets are Class I and Class III, respectively. This distribution results in DenseNet121 models biased in favor of the dominant class of each dataset in the LL setting, as illustrated in Figure 10a,b. On the other hand, the overall class percentages for the combined test sets of both datasets are 0.38, 0.29, and 0.33, respectively. Thus, the FL and CL settings demonstrate a more balanced accuracy distribution, as shown in Figure 10c,d. Class I, having the highest percentage on the combined test sets, achieves higher accuracy of 61.6% in the FL setting compared to 50% in the CL setting. Conversely, Class III shows an accuracy of 57.7% in the CL setting, which is only 32% in the FL setting. This discrepancy between the FL and CL settings across different classes highlights that the impact of data bias is more pronounced in the FL setting compared to the CL setting.

### 4.2. Comparative Analysis with Respect to the Related Works

The other studies using FL in dentistry obtained aligned performance improvement behaviors. In Schneider et al.’s study, FL outperformed LL for most participants (eight out of nine), with models trained via FL achieving better results on their respective local test datasets [7]. In contrast, Charité, with the largest dataset, managed to achieve comparable performance with LL and FL. However, all nine FL models demonstrated superior generalization across all participants when evaluated on a combined test set, highlighting FL’s advantage in generalizability over LL. This suggests that even centers with extensive datasets may benefit from FL, particularly when models are intended for broader application beyond a single institution. Additionally, Liu et al. also observed that the global model performs more effectively under imbalanced distribution compared to balanced distribution [8]. Liu et al. specifically analyzed the effect of imbalanced data amount over varying numbers of clients. However, our dataset enables us to analyze not only the imbalance in the total amount of the datasets but also the interclass imbalances within each dataset.

The performances of the some of the most prominent studies are given in Table 7. The studies of Ibragimov, Arık, Lindner and Cootes firstly detect the 19 landmarks and then calculate the ANB angle [1,9,25]. A two-phase approach like this achieves higher performance but requires the accurate annotations of 19 landmarks for each of the images of new datasets. However, the model used in this study needs only the class label of the image during training. In this work, image labeling is performed by only three landmark annotations per image, which may affect the data curation burden for big datasets in the future. Kim et al. also offered a model which directly classifies the image based on only the image class label and achieved high accuracy values on their local dataset [4,10]. Nevertheless, the CNN model offered by Kim et al. is developed and tested on our LL setting but achieved an accuracy value of 0.6162. It is difficult to offer a general solution to different image processing and machine learning tasks because of the non-linear structure of these methods and the change in the input data may result in different results. Based on this fact, the pre-experiments on DenseNet121 proposed by Nan et al. and the whole-image approach offered by Kim et al. lead this study to prefer the former one [4,24].

### 4.3. Statistical Analysis of FL Contribution

The comparison table for assessing the statistical significance by the *p*-values of the performances of the models for LL vs. FL and CL vs. FL settings are given in Table 8. All the comparisons are performed by a paired t-test after performing a five-fold cross-validation and verifying normality of the results obtained using the Shapiro–Wilk test. It is observed that applying the FL setting for DenseNet121 on the ISBI is statistically significant (*p* < 0.05), whereas there is no significant improvement for the Dicle dataset, which stems from the fact that the amount of Dicle is more than two times that of ISBI. Similarly, applying the DenseNet121_SA and DenseNet121_SPP models on the FL setting also does not show a statistically significant (*p* > 0.05) improvement on the Dicle dataset with respect to the LL setting. However, in all the other cases, applying FL shows a statistically significant (*p* < 0.05) improvement over LL.

In the last column of Table 8, it is observed that applying FL does not show a statistically significant (*p* > 0.05) performance loss compared to CL, except for the basic DenseNet121 model. The findings in Table 8 can be summarized as the effect of the FL contribution is significantly large, especially for smaller datasets like ISBI and augmented models, whereas the performance loss of FL over CL is statistically negligible for augmented models.

### 4.4. Evaluating the Labeling Procedures of Dicle and ISBI Datasets

Last but not least, there are two different angle standards for the orthodontic skeletal classification. The Steiner analysis offers 3.2–5.7 for Class 1, >5.7 for Class 2 and <3.2 for Class 3, whereas Kim et al. offer 0–4 for Class 1, >4 for Class 2, and <0 for Class 3 [1,2,3,4]. The orthodontist who labeled the Dicle dataset also used the standard offered by Kim et al. However, the Steiner analysis is used for the ISBI dataset as the previous studies used this standard [1]. It is noteworthy that FL can even mediate different labeling standards of the training and testing datasets, which may be a common case in real-world data. It is also remarkable to emphasize that this classification task is particularly difficult due to the narrow ranges–such as 3–4 degrees–among the different classes [9].

### 4.5. Limitations and Future Work

However, this study is not without limitations. The availability and diversity of data in federated learning setups can affect model performance and generalizability. Different classification problems in dentistry involving various images and labels may yield varying performance outcomes. As a future work, integrating more diverse datasets beyond ISBI and Dicle could further enrich the model’s robustness and applicability in clinical settings. The findings of this study highlight the potential of federated learning for collaborative model training in orthodontics, offering both scalability and privacy preservation. Overall, this research contributes to advancing orthodontic diagnosis and underscores the importance of collaborative efforts and advanced methodologies in dental image analysis.

## 5. Conclusions

In summary, our study demonstrates the effectiveness of federated CNN models in orthodontic skeletal classification using cephalometric images from the ISBI and Dicle datasets. Adapting and other augmentation blocks to the DenseNet121 model in a federated framework provided a more than 26% increase in accuracy against the baseline model. The performance gain of FL over LL is achieved from 0.2% to 3.73% and from 12.51% to 20.86% on the Dicle and ISBI datasets, respectively. The introduction of the Dicle dataset further enriches available resources for dental image analysis. In addition to the promising results, it is important to acknowledge the limitations of this study. This study is conducted in a controlled environment, which may not fully capture the variability present in real-world clinical settings. Future studies should consider testing the model in more diverse and dynamic clinical environments to better assess its generalizability and performance.

## Figures and Tables

**Table 1:** Data size distribution of Class I, II, and III in ISBI and Dicle datasets.

	Dicle				ISBI			
	I	II	III	Total	I	II	III	Total
Train	318	226	141	685	48	63	189	300
Test	80	55	36	171	32	26	42	100
Total	399	284	180	856	80	89	231	400
Class Ratio	0.46	0.33	0.21	-	0.2	0.22	0.58	-

**Table 2:** Comparative analysis of performances of baseline CNNs.

	Dicle		ISBI	
Model	ACC	AUC-ROC	ACC	AUC-ROC
DenseNet121	0.5400 ± 0.04	0.6684 ± 0.04	0.6122 ± 0.09	0.6293 ± 0.05
VGG_11bn	0.5179 ± 0.04	0.6369 ± 0.03	0.5968 ± 0.09	0.6414 ± 0.01
ShuffleNet	0.4623 ± 0.04	0.5069 ± 0.03	0.5839 ± 0.1	0.5219 ± 0.02
InceptionV3	0.4960 ± 0.03	0.6198 ± 0.04	0.6005 ± 0.09	0.6025 ± 0.05
AlexNet	0.5380 ± 0.03	0.6655 ± 0.03	0.6106 ± 0.1	0.6331 ± 0.05

**Table 3:** The relative performances of both basic and augmented DenseNet121 models in the CL setting.

	Dicle and ISBI	
	ACC	AUC-ROC
DenseNet121	0.5000 ± 0.01	0.6645 ± 0.02
DenseNet121_CA	0.7333 ± 0.02	0.8832 ± 0.01
DenseNet121_SE	0.7368 ± 0.01	0.8840 ± 0.01
DenseNet121_SA	0.7272 ± 0.02	0.8715 ± 0.01
DenseNet121_SA_SE	0.7345 ± 0.01	0.8788 ± 0.01
DenseNet121_SPP	0.7244 ± 0.01	0.8702 ± 0.01

**Table 4:** The comparative performance of basic and augmented DenseNet121 models in the LL setting.

	Dicle		ISBI	
	ACC	AUC-ROC	ACC	AUC-ROC
DenseNet121	0.4347 ± 0.04	0.5719 ± 0.07	0.3116 ± 0.01	0.5345 ± 0.03
DenseNet121_CA	0.6997 ± 0.01	0.8514 ± 0.01	0.5802 ± 0.04	0.7689 ± 0.03
DenseNet121_SE	0.6977 ± 0.02	0.8548 ± 0.01	0.5990 ± 0.04	0.7817 ± 0.02
DenseNet121_SA	0.7076 ± 0.01	0.8504 ± 0.01	0.5660 ± 0.03	0.7627 ± 0.02
DenseNet121_SA_SE	0.7084 ± 0.01	0.8537 ± 0.01	0.5935 ± 0.02	0.7819 ± 0.02
DenseNet121_SPP	0.6782 ± 0.01	0.8437 ± 0.01	0.4901 ± 0.05	0.7439 ± 0.01

**Table 5:** The relative performance of standard and augmented DenseNet121 models in FL setting.

	Dicle and ISBI					
	ACC	Precision	Recall	F1 Score	AUC-ROC	Cohen’s Kappa
DenseNet121	0.4367 ± 0.03	0.4101 ± 0.08	0.4045 ± 0.04	0.3784 ± 0.08	0.5529 ± 0.09	0.1061 ± 0.07
DenseNet121_CA	0.7310 ± 0.01	0.7384 ± 0.02	0.7310 ± 0.01	0.7294 ± 0.01	0.8703 ± 0.01	0.5935 ± 0.02
DenseNet121_SE	0.7340 ± 0.01	0.7464 ± 0.01	0.7340 ± 0.01	0.7352 ± 0.01	0.8784 ± 0.01	0.5964 ± 0.01
DenseNet121_SA	0.7318 ± 0.02	0.7449 ± 0.01	0.7318 ± 0.02	0.7339 ± 0.02	0.8772 ± 0.01	0.5931 ± 0.03
DenseNet121_SA_SE	0.7457 ± 0.01	0.7602 ± 0.02	0.7457 ± 0.01	0.7475 ± 0.01	0.8755 ± 0.02	0.6139 ± 0.02
DenseNet121_SPP	0.6987 ± 0.02	0.7060 ± 0.02	0.6987 ± 0.02	0.7006 ± 0.02	0.8538 ± 0.01	0.5441 ± 0.04

**Table 6:** Accuracy contributions of FL with respect to LL, and performance sacrifices compared to CL.

	Dicle	ISBI	Dicle and ISBI
	LL vs. FL	LL vs. FL	CL vs. FL
DenseNet121	0.002	0.1251	0.0633
DenseNet121_CA	0.0313	0.1508	0.0023
DenseNet121_SE	0.0363	0.1350	0.002
DenseNet121_SA	0.0242	0.1658	−0.0046
DenseNet121_SA_SE	0.0373	0.1522	−0.0112
DenseNet121_SPP	0.0205	0.2086	0.0257

**Table 7:** Performance comparison table for CL results in the literature.

Study	Dataset	Data Size	ACC
Nino-Sandoval et al. [2]	Local	229 (70% train-val 30% test)	0.6522
Ibragimov el al. [1]	ISBI	250 (60% train 40% test)	0.7664
Lindner and Cootes [1,9]	ISBI	250 (60% train 40% test)	0.7583
Arık [25]	ISBI	250 (60% train 40% test)	0.7731
Kim et al. [10]	Local	960 (85% train-val 15% test)	0.938
Kim et al. [4]	Local	1574 (92.5% train-val 7.5% test)	0.96
DenseNet121_SE	Dicle and ISBI	856 (80% train 20% test) 400 (75% train 25% test)	0.7368

**Table 8:** Measuring statistical significance by *p*-values of the accuracy contributions of the FL with respect to the LL, and performance sacrifices compared to the CL.

	Dicle	ISBI	Dicle and ISBI
	LL vs. FL	LL vs. FL	CL vs. FL
DenseNet121	0.9383	0.0001	0.009
DenseNet121_CA	0.0140	0.0001	0.8698
DenseNet121_SE	0.0117	0.0002	0.7542
DenseNet121_SA	0.0577	0.00001	0.7530
DenseNet121_SA_SE	0.0085	0.00001	0.2871
DenseNet121_SPP	0.1727	0.00006	0.0935

## Data Availability

The ISBI dataset is publicly available [1]. The Dicle dataset and ISBI skeletal classification information is publicly available at https://doi.org/10.48623/aperta.274413.
